# Effect of hydro-alcoholic extract of *Panax ginseng* and Ampicillin treatment in an animal model of *Listeria monocytogenes*-induced endocarditis

**DOI:** 10.22038/AJP.2022.21227

**Published:** 2023

**Authors:** Seyed Zanyar Athari, Zahra Karamouz, Mir Alireza Nourazar, Yousef Doustar, Younes Anzabi

**Affiliations:** 1 *Department of Physiology, Faculty of Medicine, Tabriz University of Medical Sciences, Tabriz, Iran*; 2 *Department of Basic Sciences, Faculty of Veterinary Medicine, Tabriz Medical Sciences, Islamic Azad University, Tabriz, Iran*; 3 *Department of Pathobiology, Faculty of Veterinary Medicine, Tabriz Medical Sciences, Islamic Azad University, Tabriz, Iran*

**Keywords:** Listeriosis, Cytokines, ELISA, Heart, Myocardial-edema

## Abstract

**Objective:**

Endocarditis is a rare but serious infection caused by *Listeria monocytogenes*. *Panax ginseng* demonstrated multiple immunomodulatory effects in earlier studies. Ampicillin is known as an effective antibiotic in the treatment of this disease. Therefore, this study aimed to evaluate the effect of hydro-alcoholic extract of *P. ginseng* and ampicillin treatment in an animal model of *Listeria monocytogenes*-induced endocarditis.

**Materials and Methods:**

Thirty mice, 5-7 weeks old, were randomly divided into five groups (n=6) including Healthy Control, Infected, Ampicillin (20 mg/kg, subcutaneous) treatment, Ginseng (0.025 mg/kg, intraperitoneal) treatment, and Ginseng (0.025 mg/kg, intraperitoneal) +Ampicillin (15 mg/kg, subcutaneous) treatment groups. The concentration of cytokines in heart tissue, such as IL-1 (interleukine-1), IL-6, IL-8, and TNF-α (Tumor Necrosis Factor-α), was measured. Histopathological changes were evaluated in heart tissues.

**Results:**

The levels of cytokines were significantly decreased in the Ampicillin+Ginseng treated group compared to the other experimental groups. Microscopically, pathologic changes in heart tissue were concomitant with biochemical findings, which in the infected group, neutrophils and mononuclear cells infiltration in endocardial tissue, myocardial cell necrosis, and edema were detectable. The Ampicillin+Ginseng group showed no significant changes compared to the normal control group.

**Conclusion:**

This study showed that ginseng hydro-alcoholic extract plus ampicillin has better efficacy than the extract or antibiotic alone against experimental endocarditis caused by Listeriosis.

## Introduction

Endocarditis is a rare but serious infection observed in about 8% of patients infected with *Listeria monocytogenes *(Özgenç and Meltem, 2016). In 2011, 94% of identified *L. monocytogenes *cases were hospitalized, with a case fatality rate of 13% (Valckx et al., 2017). Although listerial-induced cardiac infection is less common than *Staphylococcus aureus* or *Streptococcus Viridans*, bacterial endocarditis due to *L. monocytogenes* because of its high mortality still, needs careful examination for diagnosis and treatment (Brouqui and Raoult, 2001; Hill et al., 2006). Previous studies have shown that the myocardium is an inaccessible place for bacteria to multiply and form abscesses, while Luo et al. stated that *L. monocytogenes *has the ability to form abscesses, multiply and form colonies at this site (Luo et al., 2003). Besides, cases of bacterial myocarditis and abscess formation due to listeria infection have been previously reported (Makaryus et al., 2004; Haddad et al., 2007). 

Previous studies have shown that ampicillin is known to be an effective antibiotic in Listeriosis (Temple and Nahata, 2000). Biological materials are a branch of modern pharmacotherapy of diseases. Although there are various pharmacological agents for the treatment of various diseases, most patients are not able to tolerate the side effects of chemical drugs, and most biological substances of natural origin have very few side effects. Therefore, the use of new and complementary therapies for the treatment of infectious diseases and control of their complications, especially in developed countries, is discussed (Mohajeri et al., 2014).

Ginseng is commonly used in traditional Chinese medicine as a tonic and an adaptogen to reduce and boost the immune system (Kachur and Suntres, 2016). Ginseng is a perennial adaptogenic herb plant belonging to the genus* Panax *of the family Araliaceae (Kachur and Suntres, 2016). *Panax ginseng *has been reported to harbor a variety of bioactive chemical compounds, including polyacetylenes, alkaloids, vitamins, minerals, phenolics, flavonoids, and triterpenes (Kim and Choi, 2018). Ginseng can stimulate Natural Killer cells and T cells, produce multiple cytokines from activated immune cells, and induce anti-tumoral and antimicrobial activities in macrophages (Riaz et al., 2019). Ginseng extracts which were shown to have both bacteriostatic and bactericidal actions, seem to exert their effects by several mechanisms including disruption of biofilms, inhibition of quorum-sensing and virulence factors, and altering motility (Kachur and Suntres, 2016). Ginseng has been claimed to increase the antioxidant capability against oxidative stress (Hsu et al., 2017). 

The present study aimed to evaluate effect of hydro-alcoholic extract of *Panax ginseng* and ampicillin treatment in an animal model of *Listeria monocytogenes*-induced endocarditis.

## Materials and Methods

**Animals**

All experiments were conducted using male mice (n=30) aged 5-7 weeks old (18-22 g), purchased from the Veterinary Medicine Faculty breeding Laboratory (Veterinary Faculty, Tabriz Islamic Azad University, Iran). All animal experiments were performed with guidance and approved by Ethical Committee of Islamic Azad University, Tabriz Branch, Tabriz, Iran (IR.IAU.TABRIZ.REC.1401.090). The mice were kept in the experimental facility at 60% humidity and 23±2˚C, on a 12-hr light-dark cycle. One week after transfer to the laboratory and adapting to the environment, the mice were randomly divided into five groups of six animals each, including the Normal control group (NC) which was injected by normal saline 0.9% in intraperitoneal route, Infected group (LD) which was injected by *L. monocytogenes* suspension (1.5×10^8^ colony-forming units of bacteria in 1 ml of prepared inoculums) in i.p. route, Ampicillin treatment group (AT) which received the drug (20 mg/kg, subcutaneous) (Allen, Dowling and Smith, 2005) after the infection for one month, Ginseng treatment (GT) which received the extract (0.025 mg/kg ip) (Ahn et al., 2006) after the infection for one month, and Ginseng +Ampicillin treatment (GAT) group which was treated by ampicillin and the extract in the same manner. This treatment process was performed at the laboratory site every day at 10 o’clock.

**Preparation of Bacteria**

*L. monocytogenes *were prepared from Persian Type Culture Collection (ATCC 19114). The lyophilized bacterium was inoculated on BHI (Brain heart infusion) media (Merck, Darmstadt, Germany) broth and BHI agar media and incubated for 24–48 hr at 37°C. Three to five well-isolated colonies of the same morphological type in a plate of BHI agar media, were mixed in 10–15 ml of sterile normal saline solution. Bacterial suspension density was adjusted to 0.5 McFarland standards, which has an absorbance of 0.08–0.10 at 625 nm by UV–visible spectroscopy measurements (250–800 nm, Perkn Elmer, Germany). This is equivalent to 1.5×10^8^ colony-forming units of bacteria in 1 ml of prepared inoculums (Forbes et al., 2007).

**Preparation of ginseng** **extract**

Hydroalcoholic extract of ginseng was determined as described previously which briefly first the root of the plant is powdered and then the extract is prepared using the percolation method in which the dry powder is poured into the cylindrical part of the percolator that is filled up to two-thirds with alcohol 80% and the rest with water. When the first solution exits the end valve, the valve is shut and the extract is collected after 24 hours. Then the extract collected is dried at 30 -40°C in a sterile environment (Valipour et al., 2014). Ginseng was stored as a dried powder at 4˚C for future use. Routine quality controls of ginseng verified no contamination of endotoxin of Gram-negative bacteria or other microbial products in the procedure of ginseng preparation. Ginseng was dissolved in PBS (Phosphate-buffered saline) (Sigma-Aldrich, Darmstadt, Germany) (pH 7.4) and was filtered through 0.25µm membranes (Millipore, Bedford, MA) just before being injected into the mice (Ahn, Song, et al., 2006).

At the end of the study, the mice were anesthetized with high doses of ketamine 80 mg/kg (Alfasan, Germany) and xylazine 10 mg/kg (Alfasan, Germany). 

**Tissue sampling**

Heart tissue samples were homogenized in 10 volumes of 50 mM sodium phosphate buffer (pH 7.4) at 4°C and centrifuged at 4.500×g for 15 min, and the supernatant was collected for testing IL-1, IL-6, IL-8 and TNF-α concentrations. Commercially available rat enzyme-linked immune sorbent assay (ELISA) kits (Bioassay Technology Laboratory, Shanghai Kora in Biotech Co. China) were used to determine the cytokines concentrations according the instructions of the manufacturers (Minato et al., 2003).

**Histopathology**

A part of the hearts was used to prepare histopathological lamel, and the samples were fixed in 10% buffered formalin and embedded in paraffin. Paraffin sections (5-µm thick) were stained with hematoxylin and eosin (H&E). The tissue sections were examined by experienced pathologists using an Olympus light microscope (Olympus, Tokyo, Japan). All measurements and scoring were performed on blinded slides (Song et al., 2014). The severity of heart tissue injury in terms of cell necrosis, edema, and infiltration of inflammatory cells, was scored from zero to 4 (1- No lesion (-), 2- Mild cell damage (+), 3- Moderate cell damage (++), and 4- Severe cell damage (+++)).

**Data analysis**

A one-way ANOVA followed by Tukey post-hoc test (SPSS ver. 22) was used to test the difference between the control and treatment groups in the immunological results and the Kruskal Wallis test, followed by the Mann Whitney U test, was used to compare the degrees of pathological changes in the heart. The criterion for statistical significance was set at p*<*0.05.

## Results

**Immunological results**

**Figure 1:**
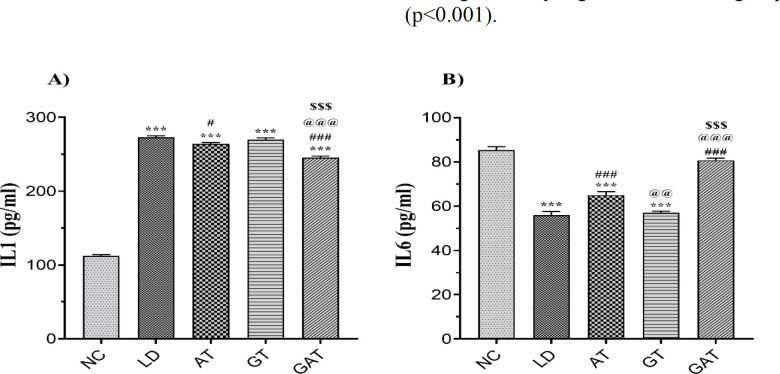
Effect of treatment on A) the level of IL-1 and B) the level of IL-6 in the experimental groups. Data are expressed as mean±SEM (n=6). ***p<0.001 vs. NC group, #p<0.05 vs. LD group; ###p<0.001 vs. LD group, @@@p<0.001 vs. AT group, $$$p<0.001 vs. GT group. [NC, Normal Control; LD, Infected; AT, Ampicillin Treatment; GT, Ginseng Treatment; GAT, Ginseng+Ampicillin Treatment; IL, Interleukin].

Figure 1-A indicates that infection with listeriosis increased IL-1 level in the LD group compared to the NC group (p<0.001). On the other hand, this cytokine decreased in the AT (p<0.05) and the GAT (p<0.001) groups compared to the LD group. The concentration of IL-1 in the GAT group was significantly lower than the LD, AT, and GT (p<0.001) groups and considerably higher than the NC group (p<0.001). Nevertheless, there was no significant difference between the GT and the LD groups regarding IL-1 level.

According to the results shown in Figure 1-B, the IL-6 level in the LD group was significantly lower than the NC group (p<0.001). This result showed that the level of this cytokine was significantly increased in the AT and GAT groups (p<0.001) compared to the LD group. There was no difference between the GAT and the NC groups in IL-6. The concentration of IL-6 in the AT (p<0.01) and the GAT (p<0.001) groups was significantly higher than the GT group. There was no difference between the GT and the LD groups in IL-6.

**Figure 2:**
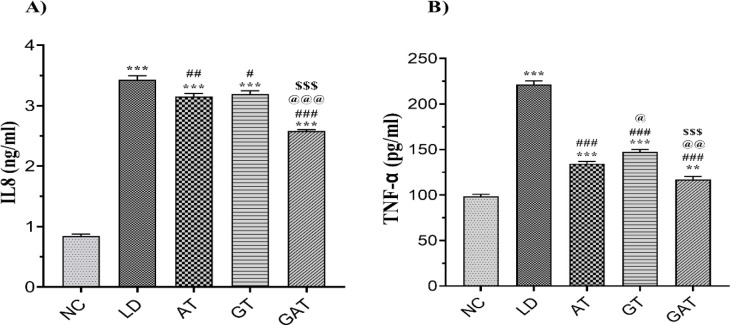
Effect of treatment on A) the level of IL-8 and B) the level of TNF-α in the experimental groups. Data are expressed as mean±SEM (n=6). ^**^p<0.01 vs. NC group; ***p<0.001 vs. NC group, #p<0.05 vs. LD group; ##p<0.01 vs. LD group; ###p<0.001 vs. LD group, @p<0.05 vs. AT group; @@p<0.01 vs. AT group; @@@p<0.001 vs. AT group, $$$p<0.001 vs. GT group. [NC, Normal Control; LD, Infected; AT, Ampicillin Treatment; GT, Ginseng Treatment; GAT, Ginseng+Ampicillin Treatment; IL, Interleukin].

As shown in Figure 2-A, the level of IL-8 in the LD group was significantly higher than the NC group (p<0.001). On the other hand, this cytokine level decreased in the AT (p<0.01), GT (p<0.05), and GAT (p<0.001) groups compared to the LD group. The concentration of IL-8 in the GAT group was significantly lower than the LD, AT, and GT (p<0.001) and significantly higher than the NC group (p<0.001). Nevertheless, there was no significant difference between the AT and the GT groups in terms of IL-1 level. The level of IL-8 in the AT and the GT groups was significantly higher than the NC group (p<0.001).

TNF-α results in Figure 2-B demonstrate a significant increase in the LD group compared to the NC group (p<0.001). This factor was significantly decreased in the AT, GT, and GAT groups compared to the LD group (p<0.001). This cytokine was significantly higher in the AT (p<0.001), GT (p<0.001), and GAT (p<0.01) groups compared to the NC group. TNF- α in the GT group was significantly higher than the AT group (p<0.05). This result showed that the level of this cytokine in the GAT group was significantly lower than the AT (p<0.01) and GT (p<0.001) groups.

**Histopathological results**

**Figure 3:**
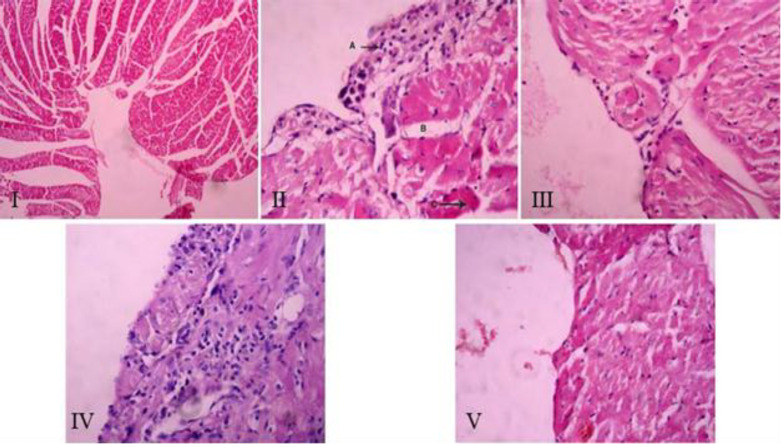
Microscopic view of the heart tissue of NC group (Hematoxylin-Eosin staining, ×20), Figure 3-II: LD group, Figure 3-III: AT group, Figure 3-IV: GT group, Figure 3-V: GAT group (Hematoxylin-Eosin staining, ×40).

**Figure 4:**
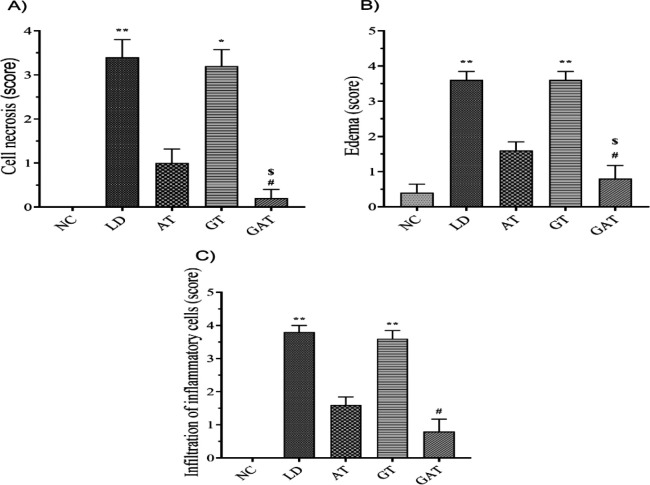
Effect of treatment on A) Cell necrosis, B) Edema, C) Infiltration of inflammatory cells in the experimental groups. Data are expressed as mean±SEM (n=6). ^*^p<0.05 vs. NC group; **p<0.01 vs. NC group; #p<0.05 vs. LD group; $p<0.05 vs. GT group. [NC, Normal Control; LD, Infected; AT, Ampicillin Treatment; GT, Ginseng Treatment; GAT, Ginseng+Ampicillin Treatment].

The histopathological findings on changes in endocardial tissue in different groups of the present study showed that no histopathological damage in distilled water injection in NC group (Figures 3-I, 4-A, 4-B, and 4-C). Also, the histopathological study (Figure 3-II) showed that after bacterial injection, in the endocardial tissue of LD group, myocardial cell necrosis (4-A), myocardial edema (4-B), and neutrophils and mononuclear cells infiltration in endocardial tissue (4-C) were detectable which was significantly higher than NC group (Figures 3-II, 4-A, 4-B, and 4-C). The microscopic view of the endocardial tissue of AT group (Figure 3-III), showed very mild infiltration of the inflammatory cells and it was fewer than in LD group (Figures 3-III, 4-A, 4-B, and 4-C). In the microscopic view of the endocardial tissue of GT group, based on the observations (Figure 3-IV) and a significant difference compared to the other groups, the infiltration of neutrophilic and mononuclear cells into the endocardium could be observed (Figures 3-IV, 4-A, 4-B, and 4-C). On the other hand, the histopathological findings of the present study on the endocardium of GAT group showed that the histological appearance of the tissue was normal, and no inflammatory changes were observed (Figures 3-V, 4-A, 4-B, and 4-C).

## Discussion

Ginsenoside, a ginseng active ingredient, has significant potential as an antibacterial agent in the treatment of bacterial Gram-positive and Gram-negative infectious diseases. Also, the mentioned compound is known as an antiseptic material. Previous studies stated that hydro-alcoholic extraction of plants results in the complete extraction of saponin, alkaloid, phenolic, and flavonoid compounds. These compounds have antibacterial, antioxidant, and anti-inflammatory properties. The present study results demonstrated that ginseng hydro-alcoholic extract showed its anti-inflammatory effects both alone and in combination with ampicillin (Sadraei et al., 2003; Sarkar and Mandal, 2012; Ashoori et al., 2020). Combined treatment with this substance and antibiotics is mainly used to extend antibacterial properties and prevent the development of drug resistance (Kim and Yang, 2018). The present study showed that levels of cytokines IL-1, IL-6, IL-8, and TNF-α were significantly increased in the LD group compared to the NC group (p<0.05), indicating inflammation in the mice of the LD group. Consistent with these results, Araújo et al. (2015) stated that in infectious endocarditis caused by *Staphylococcus aureus*, the levels of cytokines IL-1, IL-12, and TNFα in patients were higher than non-diseased groups (Araújo et al., 2015). The present study revealed that treatment with ginseng to some extent improved levels of the mentioned cytokines that were significantly different from the levels those in the NC group mice (Table 1), which is consistent with the results of the findings of Lim et al. (2002) that reported that ginseng has shown antiseptic and immune-stimulating properties by stimulation of macrophages against *Staphylococcus aureus* (Lim et al., 2002). Ahn et al. (2019) study in an experimental model of peritonitis revealed that the extract of ginseng has an anti-inflammatory effect and improves IL-1 and IL-6 which is in line with the results of the present study (Ahn et al., 2019). Also, in a study by Choi et al. (2012) on the antibacterial activity of ginseng, it was found that the ginseng extract has an anti-*Listeria* activity (Choi et al., 2012). As an explanation of the mentioned properties, it has been reported that many of the biological effects of saponins are related to their function on cell membranes (Sung and Lee, 2008). Ginseng is among the compounds mentioned by Lee et al. who found that the antibacterial activity of Ginseng was probably related to the mechanism of destruction of the bacterial membrane (Lee et al., 2013). Ginseng is well characterized to modulate the immune response via TLRs (Toll-like receptors), especially TLR4. According to previous studies, *Panax ginseng* downregulated TLR4, which induces the expression of proinflammatory cytokines like IL-1, IL-6, and TNF-α (Nakaya et al., 2004; Ahn et al., 2006). In a study, oral administration of *P. ginseng* extracts to mice was shown to attenuate increased hepatic TLR4 expression in alcoholic liver disease (Bang et al., 2014). Also, in the microscopic findings of our study, the presence of inflammatory cells, edema, and necrosis in the endocardial cells in the LD group mice were observed that represent endocarditis in the studied animals. In the AT group mice, a mild secretion of inflammatory cells was observed that shows non-elimination of cellular damage in the mentioned group. Also, treatment with ampicillin did not have a significant effect on the recovery of the listeriosis disease. Our findings are in line with the results of Ukuku and Shelef (1996) research which showed no significant effect for ampicillin on cellular damage caused by the mentioned disease (Ukuku and Shelef, 1996). The results of Sung and Lee (2008) research showed that co-administration of ginseng, kanamycin and cefotaxime increased antibacterial effect against therapeutic resistant *Staphylococcus aureus * isolated from the patients (Sung and Lee, 2008). These results are in line with the present study's finding, indicating that the treatment by ginseng extract with ampicillin drug makes a better recovery than other groups.

According to a report, a percentage of patients with listeriosis suffered from endocarditis (Özgenç and Meltem, 2016). This study was consistent with our research done in an experimental model of endocarditis in rats. As a result, it can be said that finding a treatment protocol for this complication, such as the use of ampicillin and ginseng, is effective for *L. monocytogenesis* treatment.

Hydro-alcoholic extract of ginseng with ampicillin had an ameliorating effect on endocarditis caused by *L. monocytogenesis*, and this combination had a better effect than each alone against experimental endocarditis caused by listeriosis. 

Of course, to draw a robust conclusion on this, it is necessary to do more extensive experiments by using other extracts and antibiotics.

## Conflicts of interest

The authors have declared that there is no conflict of interest.
